# The Dental Convention

**Published:** 1858-04

**Authors:** A. J. Volck

**Affiliations:** Baltimore


### ARTICLE VII.

### *The Dental Convention.*

MESSRS. EDITORS.—Having waited in vain to hear some plain spoken remarks from some one of my professional brethren upon the report of the late Dental Convention in Boston, published in the last October number of your Journal, I submit to you for insertion some thoughts to which the perusal of that report has given rise, in my own mind.

Conventions of any scientific profession, if properly conducted, must of necessity tend to elevate the profession in the eye of the world, and improve the members themselves. To accomplish these objects it is desirable that they should be organized upon as liberal a basis as the nature of the science will permit. Yet such conventions cannot possibly fulfil their purposes, if they are without system or rules. Employment in any great and good work brings with it self-respect, and if I may say so, pride for the work itself, which makes it impossible for its devotee to look with complacency and see it desecrated by intruders and tyroes, unskilled and superficial. We would look with wonder at a medical convention into which any man professing to cure, though he be only a country parson with a medicine box and "Reese's Family Physician," was admitted a member, and was allowed to take active part in the discussions. We would think little of a regularly bred physician who was so void of self-respect as to call himself a member of such a thing; yet our dental convention is even more liberal than this. They considered every man "*professing* to practice dentistry" entitled to membership, with all those who ever practiced dentistry in their lives, whatever their occupation at present may be; they invite members of other professions to join, they do away with constitution, by-laws or rules, and invite all calling themselves dentists any where in the world, be they German barbers, English barbers—surgeons, tooth-pulling country blacksmiths, or regularly bred dentists, to unite with them. It is quite indifferent to the convention, whether any one has scientific professional education or not, any one may speak, whether prepared or not, without notice, and say what he pleases, provided only he professes to have done something to teeth some time during his life.

Indeed our profession must be a poor one, to be obliged to make such liberal allowance in order to assemble a respectable number of men for such a purpose!

The consequence of all this we see in the report. Such an incongruous mixture of sense and nonsense was probably never heard of before in any so-called scientific convention. The few good things spoken on the occasion, were said by a few members who evidently attended, either expecting to meet educated professional brethren, or were urged to attend by their peculiar position in the profession as editors of our journals. Except the remarks of these gentlemen, it must be admitted, the most of the speeches were ridiculously small and vapid. Some of the members could not let such a glorious opportunity to blow their individual trumpets and to luxuriate in the incense of self-laudation, pass by, while on the whole the members showed they spoke without preparation, and sometimes without the thought which a proper regard for their reputation as sensible men would seem to demand. Nothing was said to throw a new light upon any subject under discussion, and no papers were read.

The conclusion which must be arrived at by any one who takes this body of men as a representation of the profession, is, that we are a set of mere manipulators, but by no means scientific men. I feel humbled and ashamed, when I compare this report with the reports of conventions of other professions, both in this country and abroad. Not to be personal, I abstain from quoting from the report, any one who reads it will understand my allusions.

But the most preposterous thing in the whole affair, and most humiliating confession of their imbecility, was the resolution of the convention "to collect money from members of the profession, *to pay some man to make analyses of any thing which any one of them might send him, and to conduct physiological, pathological, chemical and hygienic experiments, as connected with dentistry." !!* Shame on the men who could vote for such a resolution! Have we become so low as to be mere mechanics, leaving the scientific part of our work to some man or other who does the thinking for us for dollars and cents? Are we so weak as not to be able to think for ourselves, or do the gentlemen suppose that any man would do scientific work better for money than we can do it for our love of and interest in the science?

Really, the gentlemen who originated this grand scheme should take out a patent for it and sell it to other professions. Physicians, lawyers and others, might then pay some man to solve all knotty questions for them, and bless dentists for their ease. I consider this one of the grandest inventions of the age!

There is, then, no further need for study! Reading scientific works and using our brains are useless bores, and may be given up; henceforth, when the day's operating is done, we may sit in our easy chairs and read novels, knowing that "the man" will send us results when he gets at them. Nice results they will be, to be sure!

Such things however, we must expect, when there is such want of professional education. Of course it is not to be expected that men who never studied, should know the importance of self-thought and personal investigation.

Why dont the gentlemen who so liberally proffered their ten and fifty and hundred dollars, for such a lazy object, pay this money to our colleges, and enable them to pay professors, who are, or ought to be, the nurses of our profession, a decent salary, so as they may have a chance to conduct original investigations? Why dont these generous gentlemen furnish money for prize essays or pay for a proper education of students of talent in needy circumstances? Perhaps the gentlemen dont see the cash returns for such investments plainly enough.

It has been said by some, that practicing dentists have no time for investigation. Not considering the stupidity of such a remark, let me ask, how do dentists spend the long winter evenings, or the spare time in the hot season? We have a man in the profession who has operated as much or more than any one of us, and yet has had time to furnish us the only available dental library we possess. Would not young dentists, if they were properly reared, in the earlier years of their practice, with their education fresh upon them, and ambition for a place in the front rank, strong in them, have ample time to work for the good of the profession as well as for their own reputation?

How long will it be, before every regularly bred dentist will treat any attempt to practice without proper education, with the contempt it deserves? One gentleman in the convention was honest enough to declare himself proud of his education as a blacksmith, "(applause,)" but I have read of none who said as much for his proper education as a dentist.

I am sure that many members of the profession will coincide with me, if I say that, if these men who want to pay for information on professional matters, will apply to the editors of our journals, men in the profession will be found to answer their inquiries through the medium of these journals, not for the cash but from love to the science, and a sincere desire to promote its welfare and that of their brethren. Let those who have the ability, furnish the journals with communications on scientific subjects, so that they will not be obliged to fill up three-fourths of their pages with "selected articles," and let such men be rewarded by the respect due them as above that common herd of self-manufactured dentists and brainless quacks, that degrade our profession by their yet too great numbers.

This report has been translated into other languages, and is read and commented upon in Europe. What will their scientific men say when they read it? We have made ourselves a laughing stock; and the fear is, the term of "American dentist" will cease to be a distinction among our brethren elsewhere. Let us therefore repudiate the action of the convention as that of a handful of men, whom, as a body, the great mass of the profession do not acknowledge as their representatives. As there will probably be other conventions of the same kind, it is my request, Messrs. Editors, that you will use your influence, in future, to exclude the reporters; the members may then say what they please, and we be none the worse for it, but by all means, keep out the reporters.

Respectfully, A. J. VOLCK, D. D. S.

Baltimore, *January 26th*, 1858.

